# The National Pension Fund

**Published:** 1887-09-10

**Authors:** 


### THE NATIONAL PENSION FUND.

It will save a good deal of trouble if nurses and other hospital officials who propose joining the National Pension Fund will send their names and other particulars to *the Secretary of The Hospitals Association*, Norfolk House, Norfolk Street, W.C., and not to the Editor of The Hospital. Names continue to arrive in considerable numbers, but if those interested desire the Fund to be started before the end of the Jubilee year they must influence and interest all their friends immediately.

Readers will please note that in sending in their names, nurses and other hospital officials are not committing themselves to join any particular Fund, but simply stating their willingness to join "a Pension Fund if the rules and regulations are such as they approve."

---

The *Daily News* of September 6th, in an instructive article, which we commend to our readers, says:—"There must be at least 12,000 women engaged in nursing in the United Kingdom, and yet they have formed no sort of alliance among themselves . . . . the only attempt at combination of any sort has lately been started under the auspices of 'The Hospitals Association,' which is trying to form a National Pension Fund for trained nurses. How badly such a fund is needed few would believe who have not considered the question; yet its formation is attended with almost insuperable difficulties owing to the short time a nurse works and the small wages she receives. . . . If we seek information at any hospital, we discover . . . that the average length of her stay is about ten years, and that during that time she receives about £25 per annum, out of which she has to pay for part of her washing, besides many incidental expenses. . . . Now what sum could a nurse save during those ten years? The plums of the nursing profession—posts as matron or superintendent—are few, and the bulk of nurses cannot hope to obtain them. . . . Surely the necessity for a Pension Fund for nurses is indisputable, and if more evidence is required it can be found weekly in the pages of The Hospital, where letters in favour of the fund from matrons, sisters, and nurses are constantly being published. Nearly all of these communications express the same sentiment—the need of the fund, yet the hopelessness of getting it started without extraneous aid. Now if £50,000 of the Jubilee Offering were given to this cause, the difficulties might be considered overcome."

---

### "THE QUEEN'S BOUNTY."

Sir,—A paragraph in a weekly paper rather throws doubt on the fact that there can be any need for the application of the "Women's Jubilee Offering to the Queen," either to promote nursing or for the benefit of nurses. It appears that ignorance on the subject could alone cause such misapprehension, as in a few lines at least half-a-dozen schemes to supply special and pressing needs can be pointed out. For instance, there are many ways in which district nursing might be more widely diffused among the sick poor if more funds were available. There are numerous hospitals now in debt, whose vacant beds might be filled, if only a portion of the nurses were supported from a fund for the purpose, as they are at St. Thomas's Hospital by the Nightingale Fund.

Another branch is that of workhouse infirmary nursing, where often now one nurse may have from sixty to eighty patients under her care. Can she attend to them conscientiously? No. Can the rates be increased to furnish an extra staff? Again, no. Would not a fund to supply additional nurses similar to the "Additional Curates Fund" be a great boon in such cases?

Practical experience has proved that the poor will willingly pay a small fee to a midwife: not enough to maintain a competent person, but sufficient to prevent the patients trusting entirely to charity, which is so demoralising. If many such midwives could be partly supported by an established fund, a great want would be supplied.

These schemes are for the benefit of the community at large. To ameliorate the condition of the nurses, at least two important needs present themselves to the minds of those living amongst them.

First, a home for those who are ill—not acutely ill, so as to be fitted for a hospital ward, but weakened from fever or perhaps poisoned fingers, or strained by lifting heavy patients—needing rest and nourishing diet. There are many nurses who dread to hear the words, "You must have a holiday," for they have no place to go to. People who write glibly perhaps forget this fact, for they find to be told we must have a change, means simply to pay a round of pleasant visits; but alas! there is a very different side to the question.

Lastly, and to most minds chiefly, there is a great want of a Pension Fund. Many private nursing institutions have tried to start one for their own nurses, but in most cases have failed, and if the surplus of the Jubilee Offering should by her Gracious Majesty's decision be devoted as a nucleus for a National Pension Fund for Nurses, hundreds of women will in years to come bless its thoughtful founder.

"One who has worked in all Branches."

